# Headspace-Solid-Phase Microextraction-Gas Chromatography as Analytical Methodology for the Determination of Volatiles in Wild Mushrooms and Evaluation of Modifications Occurring during Storage

**DOI:** 10.1155/2015/951748

**Published:** 2015-04-07

**Authors:** Rosaria Costa, Selenia De Grazia, Elisa Grasso, Alessandra Trozzi

**Affiliations:** Dipartimento di Scienze del Farmaco e dei Prodotti per la Salute (SCIFAR), University of Messina, Viale Annunziata, 98168 Messina, Italy

## Abstract

Mushrooms are sources of food, medicines, and agricultural means. Not much is reported in the literature about wild species of the Mediterranean flora, although many of them are traditionally collected for human consumption. The knowledge of their chemical constituents could represent a valid tool for both taxonomic and physiological characterizations. In this work, a headspace-solid-phase microextraction (HS-SPME) method coupled with GC-MS and GC-FID was developed to evaluate the volatile profiles of ten wild mushroom species collected in South Italy. In addition, in order to evaluate the potential of this analytical methodology for true quantitation of volatiles, samples of the cultivated species *Agaricus bisporus* were analyzed. The choice of this mushroom was dictated by its ease of availability in the food market, due to the consistent amounts required for SPME method development. For calibration of the main volatile compounds, the standard addition method was chosen. Finally, the assessed volatile composition of *A. bisporus* was monitored in order to evaluate compositional changes occurring during storage, which represents a relevant issue for such a wide consumption edible product.

## 1. Introduction

Mushrooms represent an individual kingdom in the life domain. These extraordinary organisms take part in numerous aspects of human daily life: they are valuable foods, being a rich source of vitamins, proteins, and minerals, low in calories and fats [1]; sources of medicines, that is, antibiotics, and bioactive molecules, such as lectins, polysaccharides, and lanostane-type triterpenoids [2–4]; and important means in agricultural and food industries. Some edible species of mushrooms have been extensively reported in the literature concerning their chemical composition and/or their biological activity, as in the case of* Agaricus* spp. or* Tuber* spp. [5–7]. Other species, such as* Ganoderma lucidum* or* Lentinus edodes*, are pivotal medicines in the chinese tradition, for their immunomodulatory and radical scavenging actions [8, 9]. However, there is no scientific evidence for many other mushroom species which grow in the wild and are less commonly found in the groceries or vegetable markets. Nevertheless, either for a matter of taxonomic characterization or for toxicological purposes, it seems important as well to investigate the chemical composition of wild mushroom species. Although it cannot replace genetic studies, chemistry is a valid support to the morphological and physiological identification of mushroom species. For instance, some* Agaricus* species, including* A. placomyces* and* A. pseudopratensis*, which are quite similar to the well-known edible* A. bisporus*, have been demonstrated to possess gastrointestinal toxicity due to a high phenol content [10]. In this study, freshly picked up samples of ten different species of wild mushrooms from South Italy were investigated by means of headspace-solid-phase microextraction-gas chromatography (HS-SPME-GC). In addition, in order to evaluate the potential of this methodology for quantification of mushroom volatiles, samples of the cultivated species* Agaricus bisporus* were analyzed. The choice of this mushroom was dictated by its ease of availability in the food market, due to the consistent amounts required for SPME method development. Previous studies directed towards the shelf life modifications undergone by* A. bisporus* focused on (i) type of packaging material under modified atmosphere [11]; (ii) use of biobased (i.e., wheat gluten) packaging to preserve the mushroom freshness [12]; (iii) hyperspectral imaging applied to mushrooms differently packed [13]; (iv) mushroom processing through irradiation [14].

As it is well known, solid-phase microextraction (SPME) is a solvent-free sample preparation technique, introduced more than 20 years ago, which reflects the new trend toward miniaturized techniques [15]. SPME can be undisputedly considered as an environmentally friendly technique, offering a good compromise between selectivity and sensitivity, cost, and easiness of use. However, in SPME, the process of extraction is based on the achievement of equilibria between sample matrix and headspace and between headspace and fiber coating. SPME extraction is considered complete when the equilibria are established, although this phase does not correspond to the exhaustion of analytes from the sample matrix. This issue makes somehow challenging calibration procedures when SPME is chosen as a sample preparation methodology. In order to make quantitation of SPME extracted analytes, a variety of calibration procedures are available to the analyst [16]. In this context, the standard addition method has been chosen as the most suitable to the quantification of volatiles in* A. bisporus*. Due to the complexity of the matrix, only predominant components have been calibrated. Once the ruggedness of the method developed is tested, a series of samples stored under different conditions have been evaluated, in order to monitor possible changes of the volatile fingerprint or formation of off-flavours.

## 2. Materials and Methods

### 2.1. Samples

Wild mushrooms were kindly donated by the “Centro di Cultura Micologica” of Messina and belonged to the following species:* Tricholomopsis rutilans* (Schaeff.) Singer;* Agrocybe aegerita* (V. Brig.) Fayod;* Clitocybe odora* (Bull.) P. Kumm.;* Agaricus xanthodermus* Genev.;* Cantharellus cibarius* Fr.;* Clathrus ruber* Mich. ex Pers.: Pers.;* Omphalotus olearius* (DC.) Singer;* Lactarius deliciosus* (L.) Gray;* Lactarius chrysorrheus* Fr.; and* Ganoderma resinaceum* Boud. All the mushrooms were collected in Sicily (South Italy), in the bush of the Peloritani and Nebrodi mountains, during the fall of 2013-2014. Upon collection, due to the high perishability of most mushrooms, samples were immediately brought to the laboratory and analyzed. Prior to extraction, samples were chopped and approximately 3 g was placed in 20 mL crimped vials for SPME extraction. Each sample was extracted by SPME in triplicate. Mushrooms of the species* Agaricus bisporus* were purchased in local supermarkets and immediately analyzed as well. Before analysis, they were ground in an electric grinder and approximately 3 g was placed in 20 mL crimped vials for compounds extraction. Each sample was added with 10 mL of white mineral oil for SPME extraction. This is in order to have the same sample matrix conditions for both unspiked and spiked samples. For the evaluation of flavor modifications occurring during storage, some fresh samples were divided into two groups: the first group was analyzed immediately upon receipt; the second group was stored in the refrigerator at +6–8°C for 10 days.

### 2.2. SPME Extraction

SPME extraction was carried out in the headspace mode by means of an AOC-5000 autosampler (Shimadzu) hyphenated with the GC-MS system. Two different fiber coatings were tested: a 65 *μ*m polydimethylsiloxane/divinylbenzene (Supelco, Milan, Italy), 1 cm long, and a 50/30 *μ*m DVB/Car/PDMS, 1 cm long. After SPME method development, the PDMS/DVB fiber was chosen to extract the volatile components from the mushrooms. The fiber was conditioned following the manufacturer's instructions before its use. Samples were conditioned for 10 min at 50°C and then underwent the extraction step for 30 min at 50°C. Analytes were then desorbed for 1 min at 250°C in the GC injector in splitless mode. For calibration, the following standard compounds have been used: 3-octanone, 3-octanol, (2E)-octenol, benzyl alcohol, and benzaldehyde, all supplied by Sigma-Aldrich (Milan, Italy). Stock solutions of standard compounds (100,000 ppm) were prepared in white mineral oil. Serial dilutions from this stock solution were made and for each dilution 10 mL was drawn and added to the real sample. Calibration graphs were built up on 3 to 5 points, each corresponding to 3 replicates.

### 2.3. Gas Chromatography

GC-FID: a* GC-2010* system (Shimadzu), with an SLB-5ms column (Supelco/Sigma-Aldrich, Bellefonte, PA, USA), 30 m × 0.25 mm I.D. × 0.25 *μ*m d_f_, was used. For* A. bisporus* analysis, a different column stationary phase was used: Supelcowax-10, 30 m × 0.25 mm I.D. × 0.25 *μ*m d_f_. Oven temperature program was 40°C at 5°C/min to 250°C, held for 5 min and at 10°C/min to 280°C, held for 5 min. Injection mode was splitless, after 1 min split ratio: 1 : 20. Injector and FID temperatures were 250°C and 280°C. Carrier gas was He, at 30.0 cm/s (97.4 kPa). Data is processed by* GCsolution* software (Shimadzu).

GC/MS: a* GCMS-QP2010* system (Shimadzu) was used, equipped with the same columns used for GC-FID analyses. Oven program and injection parameters were the same as those for GC-FID. Carrier gas was He, at 30.0 cm/s. MS: interface and source temperatures were 250°C and 200°C; EI was 0.94 kV; mass range was 40–350* m/z*; scan speed was 1,666 amu/s, and scan interval was 0.25 s. Data handling was by* GCMSsolution*, ver. 2.51 (Shimadzu). The system was equipped with commercial (Wiley, NIST08) and dedicated mass spectral databases [17, 18]. Identification of analytes was based on three tools: (i) mass spectral matching with reference spectra; (ii) coinjection with standard compounds, in consideration of the library used, FFNSC 2, which was created in the same laboratory and with the same instrumentation utilized for this study; (iii) comparison of experimental retention indices with published values. For the measurement of retention indices, a mix of *n*-alkanes ranging from heptane to triacontane (Supelco/Sigma-Aldrich, PA, USA) was injected apart.

## 3. Results and Discussion

### 3.1. Wild Mushrooms

More than one hundred compounds were determined in total in all the mushroom species investigated, as can be seen in Table 1. Some of them showed characteristic profiles, sometimes compatible with their morphological/physiological aspects. In general, the highest number of identified compounds was found in the species* T. rutilans* and* A. xanthodermus*. Characteristically,* T. rutilans* presented consistent amounts of the azulenes daucene (19%) and isodaucene (38%), peculiar compounds of carrot seed essential oil. Among the other volatiles, the characteristic presence of alpha-selinene was detected. The amber note of this component could justify the slight wood notes of* T. rutilans*.* A. xanthodermus *showed a volatile composition dominated by C8 compounds, that is, 1-octen-3-ol (82%) and 3-octanone (10%), which are secondary metabolites of most mushrooms and give them their typical odour.* Lactarius deliciosus* and* Lactarius chrysorrheus* have quite similar morphological features, with a basic difference laying on the fact that* L. deliciosus* is edible, whereas* L. chrysorrheus* is poisonous [21]. The SPME-GC analysis highlighted noticeable differences between the two species:* L. deliciosus*, which grows only under pine trees, presented, apart from an abundant fraction of 3-octanone, consistent amounts of terpenoids, such as limonene (5%), linalool (8%), and dihydrocitronellol (4%); on the other hand, in* L. chrysorrheus*, no terpenes were determined, while the characteristic C8 compounds predominated at ratios different from the edible* L. deliciosus*: 1-octen-3-ol (10%), 3-octanone (2%), 3-octanol (53%), and (2E)-octenal (5%). Also, considerable amounts of 3-methylbutanal (11%) and hexanal (2%), which are typical volatiles of truffles, were found as well.

The mushroom* A. aegerita*, when chopped or injured, smells like wine and acidic. The analysis revealed consistent amounts of ethanol (34%) and isopropyl acetate (10%); furthermore, 30% of isopentanol was found, olfactively described as having “whiskey” notes [22].

Some lethal accidents have been registered for ingestion of* Omphalotus olearius*, which can be disguised with the edible species* Cantharellus cibarius*.* O. olearius* is strong orange-coloured and contains luciferins which make it visible even in the dark, under enzymatic action of luciferase. Some azulenes have been found in the present study, although carotenoids are very likely the true responsible for the orange colour [23]; of course, carotenoids have high molecular masses and cannot be assessed by means of GC techniques. The accidental ingestion of* O. olearius* is made even worse by its appealing flavor, which recalls the floreal bouquet of wine, with mushroom notes, due to the presence of isobutanol (11%), linalool (29%), and 3-octanone (11%). On the contrary,* C. cibarius* is one of the most appreciated among edible mushrooms. Predominant volatiles of this mushroom resulted to be the C8 compounds; however, it possesses olfactive notes of peach peel and fruity candies, which are explained by the determination of hexanal, chemical utilized in food industry for the production of fruity flavors, and of (3Z)-hexen-1-ol, which smells like fruits. The mushroom* Clitocybe odora* showed a predominant amount of 5-hexen-2-one, compound with ethereal/floral notes. Upon injury, the fresh individual releases an anise-like odour and, although in low quantity (0.5%), estragole was found among volatiles. Surprisingly, no relevant amounts of C8 compounds were detected in this species.

It is worthwhile discussing apart the last two species of wild mushrooms under investigation, which were* Clathrus ruber* and* Ganoderma resinaceum*.* C. ruber* is a rare species, endemic of the Mediterranean area, with an odd look. The fruiting body, originally included in an egg, has the shape of a round ball composed of interlaced branches; the branches are spongy, red, pink, or orange, with a slime on their inner surfaces. This mushroom emits a foul, corpse-like, and pungent smell, which acts as attractant for flies in order to spread the mushroom spores. In the analyses carried out here, characteristic compounds of* C. ruber* were furfuryl methyl sulfide (68%), which is olfactively described as being pungent, sulfuraceous, radish-mustard; pentanal (15%); and camphor (5%), which are pungent as well. About the corpse-like smell, this is normally attributed to biogenic amines, such as cadaverin and putrescine, which can be detected with different analytical methodologies rather than SPME-GC-MS. Finally, samples of* Ganoderma resinaceum* were analyzed, which is a wood-decaying mushroom, from the same family of* Ganoderma lucidum*, known as Reishi in traditional chinese medicine. As indicated by the name, this mushroom has a resinaceous texture; it is not as perishable as the other wild mushrooms, due to a very low content of water. Major compounds determined in this species were hexanal and terpenes, that is, limonene and alpha- and beta-pinene. The typical C8, mushroom smelling compounds, were detected only at trace or low level (3-octanone: 3%).

### 3.2. Quantification of* A. bisporus* Volatiles

**Figure 1:**
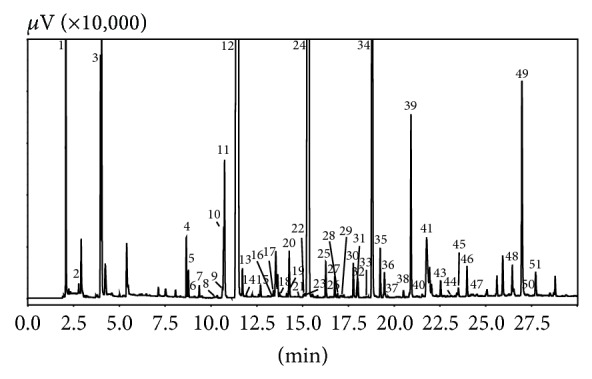
HS-SPME-GC-FID chromatogram of a fresh* A. bisporus* sample. Peak top numbers refer to Table 2.

**Figure 2:**
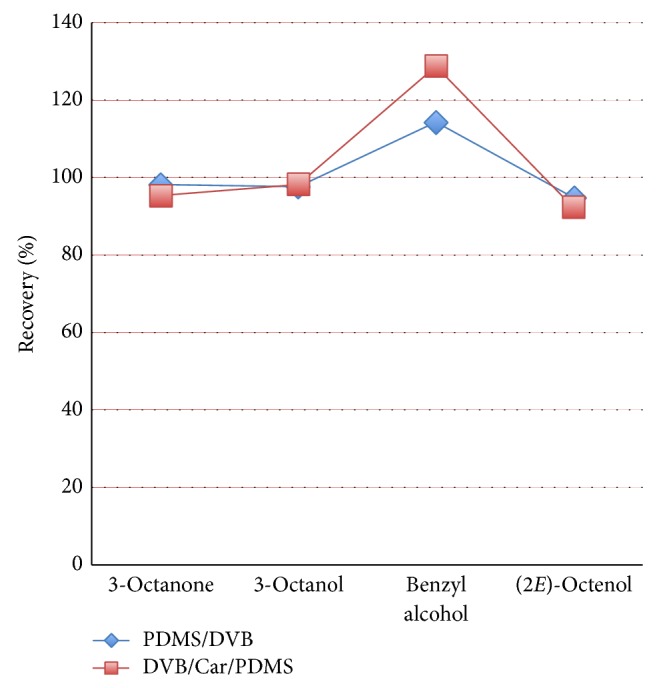
Comparison of the recoveries obtained with the SPME fiber coatings DVB/Car/PDMS and PDMS/DVB.

Table 2 reports all the compounds determined in the volatile fraction of* A. bisporus*. The correspondent chromatogram is shown in Figure 1. In total, 51 compounds were identified, with the main ones being aliphatic alcohols and ketones, typically characterized by 8 carbon atoms skeleton, such as: 3-octanone, 3-octanol, and (2E)-octenol. Also, compounds with aromatic ring were determined at considerable amounts, such as benzaldehyde and benzyl alcohol. As reported above, during SPME method development, two different fiber coatings were tested, namely, DVB/Car/PDMS (50/30 *μ*m) and PDMS/DVB (65 *μ*m). The latter was chosen in the final optimized conditions because it gave a better performance in terms of linearity and repeatability.  Table 3 reports, among other values, the linear ranges observed for both of the two fibers. As can be seen, the triple phase showed scarce linearity compared to the PDMS/DVB phase. Although the recoveries were good also for the triple phase (see Figure 2), its analytical behaviour was in general estimated as worse, basically due to carry-over and displacement effects. To solve carry-over, continuous clean-up (with hot temperature) was necessary in between of consecutive analyses, whereas the displacement effect caused dramatic reduction of repeatability and linearity. These undesired phenomena were sometimes reported as being dependent on the inner porous layer of Carboxen present in the DVB/Car/PDMS fiber coating [24]. Additionally, the triple phase showed a shorter lifetime compared to the double phase; this is reasonable, when considering the complex nature of the matrix and the weakness acquired by the triple phase by the repetitive clean-up cycles. Prior to calibration, some considerations were made. When SPME is chosen as sample preparation technique, the decision of which approach is the most convenient depends on sample matrix (liquid or solid), its complexity, and extraction mode (headspace or immersion). As mushroom flavour is being a complex sample from a solid matrix, the standard addition approach has been chosen in this study for quantification of analytes [16]. In fact, a different approach, such as external standard calibration, would not take into account the so-called “matrix effect.” Previously, the external standard method was chosen for the calibration of SPME extracts of another species of mushroom [25]. However, in that calibration procedure, the target analyte was quantified separately, without considering the solid matrix effects that affect the SPME extraction process. In fact, when target analytes are embedded in a complex matrix, they establish several uncontrollable interactions with other constituents. Furthermore, the linear regression equation obtained for 1-octen-3-ol was indiscriminately used for all the volatiles extracted, sulphur compounds included. On the other hand, the internal standard method is mostly advised for simpler matrices. The calibration procedure used in this application succeeded in the absolute quantification of mushroom key compounds. As can be seen in Table 3, satisfactory coefficients of regression were obtained, although, in some cases (e.g., 3-octanol), the procedure was time-consuming. The method was validated in terms of linearity, limits of detection, limits of quantification, recovery, and repeatability. Linearity was assessed through the construction of a multipoint calibration curve, at five different concentration levels. Three runs were carried out for each point of the curve. For the calculation of LODs and LOQs, five replicates of unspiked samples were run. LOD was measured based on the formula LOD = *t*_99_∗*s*, where *t*_99_ is the Student's *t* value relative to a 99% level of confidence and *n* − 1 degrees of freedom and *s* is the standard deviation. LOQ was measured as 10 times the standard deviation used for LOD. For recovery measurements, samples spiked with 10 ppb of standard compound were subjected to SPME extraction, each in triplicate. The detector signals corresponding to the analytes in unspiked samples were taken into account and subtracted in recovery analyses.

The GC method precision was rated as good, in terms of repeatability, through the measurement of RSD%, which was generally lower than 12%. However, the standard addition method resulted was quite troubling for benzaldehyde, compound that showed very high affinity for both of the fiber coatings tested. This means that the relation between analyte concentration and FID response, from a certain concentration downwards, was not linear any longer, making benzaldehyde quantification not reliable. Previously, an alternative methodology, namely, multiple headspace-solid-phase microextraction, was successfully applied to the quantification of truffle's volatiles [26]. As quantitative analysis in SPME is being a challenging task, this work aimed to explore further the various possibilities available to the analyst.

### 3.3. Flavor Changes during Storage

**Figure 3:**
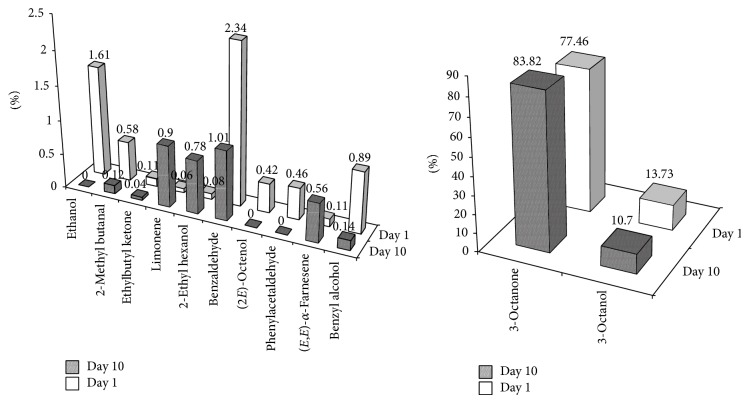
Comparison of the volatile fingerprints of* Agaricus bisporus* mushrooms. Day 1: fresh samples. Day 10: samples from a 10-day storage period in refrigerator (+6°C). Data are expressed as FID peak area % and are relative to different batches of samples (*n* = 3), each analyzed in triplicate (*i* = 3).

Once the chemical composition of mushrooms flavor is assessed, the method was applied to the investigation of possible modifications occurring during storage. In Italy, such a type of mushrooms, when purchased in the supermarkets, is commonly found in refrigerated counters. Therefore, 10-day-old mushrooms, kept in the refrigerator, were analyzed and quali-quantitative differences have been evaluated, through comparison with the profiles of fresh individuals (see Figure 3). Basically, after a period of 10 days of storage, a reduction of the volatile fraction, consisting of about 3.5%, was observed. For instance, the peaks relative to ethanol, (2E)-octenol, and phenylacetaldehyde could not be detected any longer in the stored mushrooms. A drastic decrease of compounds with aromatic ring was also observed. More or less constant was the amount of terpenoids. Interestingly, concerning the typical mushroom-smelling C8 compounds, 3-octanol and (2E)-octenol underwent a reduction. In the latter case, the amount was completely zeroed, whereas a correspondent increase of 3-octanone was observed. It seems reasonable that the two alcohols oxidize to ketone during storage.

Other researchers have previously reported the biosynthetic pathways occurring in mushrooms responsible for 8-carbon volatiles formation [25–28]. It has been demonstrated that linoleic acid, a constituent of mushrooms, is a common precursor of such molecules, utilized by certain enzymes as starting substrate [29]. More specifically, Combet et al. have shown that the formation of C8 compounds from mushroom tissues is proportional to the “damage level” that takes place during sample preparation [27]. Various papers have reported different quantities of C8 molecules, depending on the extraction methodology used (e.g., cut versus homogenized). It seems very likely that linoleic acid and enzymes are “stored” in different cell compartments of the mushroom, and they come in contact after damaging the tissues [26]. Also, the production of 1-octen-3-ol and related compounds seems to be hindered by mushrooms processing such as irradiation [14]. The latter is a technique used for destroying microorganisms and insects present in food or for improving its functional properties. Although other sophisticated techniques are commonly used for determination of shelf life or evaluation of storage conditions, the findings shown in this study demonstrate that SPME-GC-MS is a valid and feasible technique as well, to reach this aim [30].

## 4. Conclusions

Ten different varieties of mushrooms from the wild, which appear to be underinvestigated, have been analyzed for the determination of their volatile fingerprints by means of SPME-GC-MS. Considerations were given about the relationships of the chemical composition and organoleptic properties. Furthermore, a quantitative SPME method has been applied to the analysis of volatiles released by the cultivated mushroom* A. bisporus*. Once again, eight-carbon molecules have demonstrated to be key compounds in these organisms' volatile fraction. Their formation seems to involve a unique fungal biochemical pathway, reported in literature as strictly connected to lipid and fatty acid metabolism. In this context, the present study aimed to give a contribution, from the chemical point of view, to understand the highly specific biological systems of mushrooms. Biochemical “traceability” becomes very relevant when considering the fact that* A. bisporus* cultivated mushrooms are a highly perishable food; therefore, knowledge of storage modifications can improve the technology for preserving their sensory and texture qualities over time.

It is worthwhile pointing out that* A. bisporus* is the most common mushroom that can be found in the vegetable counter of a supermarket. For this reason, it seems useful to exploit the potential of SPME to detect which kind of biochemical modifications occur in the mushrooms, after harvesting and until consumption.

## Figures and Tables

**Table 1:** Relative percentages (% normalized FID peak areas) and linear retention indices of volatiles determined in wild mushrooms by means of HS-SPME-GC-MS.

Number		LRI_exp⁡_	LRI_lib_	*T. rutilans *	*A. aegerita *	*C. odora *	*A. xanthodermus *	*C. cibarius *	*C. ruber *	*O. olearius *	*L. deliciosus *	*L. chrysorrheus *	*G. resinaceum *
				Area%	Area%	Area%	Area%	Area%	Area%	Area%	Area%	Area%	Area%
1	Ethanal	—	408^c^	—	—	—	0.26	0.1	—	—	5.63	4.92	2.01
2	Ethanol	—	463^c^	—	33.88	0.17	—	—	—	—	—	—	—
3	5-Hexen-2-one	—	—	—	0.44	63.46	0.22	1.49	—	—	4.44	2.54	—
4	2-Butanol	—	603^a^	—	—	—	—	—	5.42	—	—	—	—
5	Ethyl acetate	—	606^b^	0.67	—	—	—	—	—	—	—	—	—
6	2-Methylpropan-1-ol	—	621^a^	—	13.01	—	—	—	—	—	—	—	—
7	Methyl propanoate	—	634^b^	0.64	—	—	—	—	—	—	—	—	—
8	Isopropyl acetate	—	650^a^	—	9.81	—	—	—	—	—	—	—	—
9	Isobutanol	—	650^b^	—	—	—	—	—	—	15.49	2.48	—	—
10	2-Methyl butanal	—	654^b^	4.79	—	—	0.88	—	—	—	1.28	—	—
11	2-Methyl isovaleraldehyde	—	658^b^	2.98	—	—	—	—	—	—	—	—	—
12	Butyraldehyde	—	662^a^	—	—	0.49	—	0.07	—	—	—	—	—
13	3-Methyl butanal	—	664^a^	—	—	2.63	0.23	1.32	—	—	—	—	—
14	3-Methyl-2-butanol	—	671^b^	0.02	—	1.89	0.35	0.24	—	—	—	—	—
15	Hydroxyacetone	—	698^c^	—	—	—	0.79	0.67	—	—	—	—	—
16	Pentanal	700	704^b^	—	—	—	0.73	—	14.85	—	—	—	—
17	3-Methyl-1-butanol	732	731^b^	—	29.37	21.51	0.11	2.20	1.92	4.45	—	—	—
18	2-Methyl-1-butanol	733	731^a^	—	—	—	—	—	—	0.63	—	—	—
19	2-Methyl pentanal	741	758^a^	—	—	—	0.02	0.47	—	—	—	—	—
20	Pentanol	766	762^b^	—	—	—	0.21	0.79	—	0.16	—	—	—
21	(3Z)-Hexenal	790	797^b^	0.04	—	—	—	—	—	—	—	—	—
22	Hexanal	801	801^b^	—	—	0.17	0.39	3.10	—	49.56	—	2.31	37.35
23	Ethyl butanoate	813	802^b^	0.07	—	—	—	—	—	—	—	—	—
24	(3Z)-Hexen-1-ol	845	850^b^	—	—	0.20	—	9.78	—	—	0.84	0.90	—
25	(2E)-Hexenal	852	846^b^	—	—	—	0.55	—	—	—	—	—	—
26	Hexanol	870	867^a^	—	0.14	0.14	0.52	0.39	—	6.86	—	—	4.82
27	3-Heptanone	883	885^a^	—	—	—	—	0.50	—	—	—	—	—
28	2-Heptanone	889	889^b^	—	—	4.63	—	—	—	—	2.63	—	0.50
29	(2E,4E)-Hexadienal	890	914^a^	—	—	—	—	0.02	—	—	—	—	—
30	Heptanal	902	902^d^	—	0.02	—	0.09	0.84	—		—	0.19	0.55
31	Butyl propanoate	907	907^b^	—	—	—	—	—	—	—	—	—	0.65
32	Pentyl acetate	915	912^a^	—	—	—	—	—	—	—	—	—	1.65
33	*α*-Thujene	925	924^b^	—	—	0.30	—	—	—	—	—	—	0.19
34	*α*-Pinene	932	932^b^	—	—	0.20	—	—	0.08	—	—	—	0.24
35	Ethyl tiglate	937	939^b^	—	—	—	—	—	—	—	—	—	0.85
36	Camphene	948	954^d^	—	—	—	—	—	0.14	—	—	—	—
37	(2E)-Heptenal	955	956^a^	—	—	—	0.03	—	—	—	—	—	—
38	Benzaldehyde	961	952^b^	—	—	0.07	0.06	—	—	—	—	0.09	—
39	Heptanol	970	970^a^	—	—	—	0.11	—	—	—	—	—	—
40	*β*-Pinene	976	974^b^	—	—	0.14	—	—	1.54	0.62	—	—	2.75
41	1-Octen-3-one	978	972^b^	0.94	0.12	—	0.03	2.88	—	—	—	0.26	—
42	*endo*-2-Norborneol	980	982^b^	—	—	—	—	—	0.62	—	—	—	—
43	1-Octen-3-ol	981	979^d^	4.24	—	—	82.56	—	—	1.07	—	10.49	—
44	3-Octanone	983	979^b^	3.44	9.51	0.69	10.63	21.81	—	16.06	59.80	2.56	3.15
45	Myrcene	988	988^b^	1.44	—	—	—	—	—	—	—	—	3.69
46	2-Penthylfuran	989	984^b^	—	—	—	0.11	—	—	—	—	—	—
47	1,3,5-Trimethyl benzene	994	994^b^	—	—	—	—	—	—	—	—	—	0.61
48	Furfuryl methyl sulfide	988	998^a^	—	—	—	—	—	68.52	—	—	—	—
49	3-Octanol	999	999^a^	—	0.74	0.52	—	15.85	—	1.08	—	64.16	—
50	Octanal	1005	1006^a^	—	0.01	—	0.13	0.79	—	—	—	0.70	1.78
51	*α*-Terpinene	1014	1014^b^	—	—	—	—	—	—	—	—	—	0.16
52	1,4-Cineole	1015	1012^b^	—	—	—	—	—	—	—	—	—	0.09
53	p-Cymene	1023	1027^b^	0.30	0.04	0.10	0.02	—	—	—	—	—	1.50
54	Limonene	1030	1030^a^	4.41	0.16	0.66	—	1.15	—	0.52	5.10	3.10	29.08
55	(E)-*β*-Ocimene	1044	1041^d^	0.03	0.13	0.08	—	—	—	0.13	—	—	0.79
56	*γ*-Terpinene	1057	1054^b^	0.18	—	0.40	—	—	—	0.55	—	—	2.33
57	*trans*-2-Undecenol	1057	1058^d^	—	—	—	0.07	—	—	—	—	—	—
58	(2E)-Octenal	1059	1059^a^	—	—	—	0.10	9.69	—	—	—	5.10	—
59	(2E)-Octen-1-ol	1067	1067^a^	—	0.03	—	0.11	25.85	—	—	—	1.91	—
60	*trans*-Linalool oxide	1070	1084^b^	—	—	—	—	—	—	0.27	0.44	—	—
61	cis-3-Nonen-1-ol	1071	1063^b^	—	—	—	0.01	—	—	0.13	—	—	—
62	cis-Sabinene hydrate	1072	1069^a^	—	—	—	—	—	—	—	0.89	—	—
63	Octanol	1073	1063^b^	—	2.53	—	0.33	—	—	—	—	—	—
64	p-Mentha-2,4(8)diene	1084	1088^d^	—	—	—	—	—	—	0.05	—	—	—
65	Terpinolene	1086	1089^d^	0.06	—	0.16	—	—	—	—	—	—	0.43
66	Linalool	1097	1095^b^	1.18	—	—	—	—	—	2.09	8.13	—	—
67	Nonanal	1106	1101^d^	0.51	—	—	0.03	—	1.48	—	3.76	0.77	2.47
68	Phenethyl alcohol	1116	1113^a^	—	0.05	—	—	—	—	—	—	—	—
69	Camphor	1146	1149^a^	—	—	—	—	—	4.87	—	—	—	—
70	Phenol	1166	1171^a^	—	—	—	—	—	0.07	—	—	—	—
71	(6Z)-Nonen-1-ol	1176	1173^a^	—	—	—	—	—	0.13	—	—	—	—
72	neo-Verbanol	1180	1185^a^	—	—	—	—	—	—	—	—	—	0.67
73	m-Cymen-8-ol	1185	1176^b^	—	—	—	—	—	—	—	—	—	0.14
74	Pelargol	1200	1200^a^	—	—	—	—	—	—	—	3.71	—	—
75	Estragole	1201	1201^a^	—	—	0.53	—	—	—	—	—	—	—
76	Decanal	1202	1201^b^	—	—	—	—	—	—	—	0.30	—	0.40
77	Carvone	1245	1246^a^	—	—	—	—	—	—	—	—	—	0.09
78	Linalool acetate	1249	1254^b^	—	—	—	—	—	—	—	—	—	0.60
79	2-Undecanone	1302	1293^b^	0.10	—	—	0.29	—	—	—	—	—	—
80	1-Hepten-3-ol	1323	1335^a^	—	—	—	—	—	—	—	0.21	—	—
81	Daucene	1382	1380^b^	18.74	—	0.44	—	—	—	0.09	—	—	—
82	Tetradecene	1391	1388^b^	—	—	—	—	—	—	—	0.09	—	—
83	*β*-Elemene	1392	1389^b^	0.55	—	—	—	—	—	—	—	—	—
84	*β*-Longipinene	1400	1400^b^	0.48	—	0.05	—	—	—	—	—	—	—
85	Longifolene	1412	1407^b^	—	—	—	—	—	—	—	0.16	—	—
86	*α*-Santalene	1419	1416^b^	—	—	—	—	—	0.02	—	—	—	0.46
87	(E)-Caryophyllene	1421	1417^b^	—	—	—	0.03	—	—	—	—	—	—
88	*β*-Copaene	1432	1430^b^	—	—	—	—	—	—	—	0.11	—	—
89	Cubeb-11-ene	1450	1445^d^	0.32	—	—	—	—	—	—	—	—	—
90	(E)-*β*-Farnesene	1452	1454^b^	0.48	—	—	—	—	—	—	—	—	—
91	*ε*-Muurolene	1465	1455^d^	0.31	—	—	—	—	—	—	—	—	—
92	9-epi-Caryophyllene	1468	1464^b^	0.50	—	—	—	—	—	—	—	—	—
93	*γ*-Gurjunene	1478	1475^b^	0.74	—	—	—	—	—	—	—	—	—
94	*γ*-Curcumene	1483	1479^b^	0.14	—	—	—	—	—	—	—	—	—
95	*β*-Bergamotene	1484	1483^a^	—	—	—	—	—	0.04	—	—	—	—
96	*γ*-Muurolene	1487	1483^d^	0.13	—	—	—	—	—	—	—	—	—
97	*β*-Selinene	1491	1486^d^	1.94	—	—	—	—	—	—	—	—	—
98	*α*-Selinene	1500	1501^a^	6.85	—	0.24	—	—	—	—	—	—	—
99	Germacrene A	1505	1503^d^	0.65	—	—	—	—	—	—	—	—	—
100	*β*-Bisabolene	1510	1505^b^	0.74	—	—	—	—	0.11	—	—	—	—
101	Alaskene	1515	1515^a^	1.50	—	—	—	—	—	—	—	—	—
102	(Z)-*γ*-Bisabolene	1524	1514^b^	0.38	—	—	—	—	—	—	—	—	—
103	Isodaucene	1530	1504^a^	38.41	—	0.14	—	—	—	0.19	—	—	—
104	*α*-Cadinene	1546	1537^b^	0.15	—	—	—	—	—	—	—	—	—
105	Hedycaryol	1552	1546^b^	0.12	—	—	—	—	—	—	—	—	—
106	(E)-Nerolidol	1562	1561^a^	0.82	—	—	—	—	—	—	—	—	—

LRI_exp⁡_ = experimental linear retention index measured on SLB-5ms column.

LRI_lit_ = linear retention index, retrieved from database/literature. Sources: ^a^FFNSC 2 [17]; ^b^Adams 4th edition [18]; ^c^Nist WebBook [19]; ^d^Joulain and König [20].

**Table 2:** Flavor volatiles in fresh samples of *A. bisporus* expressed as relative FID peak areas. In parentheses, quantitative values obtained by the standard addition method. Compounds are listed according to the elution order on Supelcowax-10 (PEG) column.

Peak No.	Compound	Area%
1	Ethanol	1.61
2	Butane	0.08
3	2-Methyl butanal	0.58
4	2-(1-Ethylpentyl-)-1,3-dioxolane	0.14
5	Ethylbutyl ketone	0.11
6	*n*-Undecane	0.06
7	Limonene	0.06
8	2-Butanol	<0.01
9	Ethyl hexanoate	0.18
10	2-Methyl butanol	0.15
11	Isopentyl alcohol	0.28
12	3-Octanone	77.46 (**5,587.00 *μ*g/Kg**)
13	*n*-Pentanol	0.08
14	*n*-Dodecane	<0.01
15	1-Octen-3-one	0.03
16	3-Heptanol	0.01
17	(2E)-Heptenal	0.03
18	6-Methyl-5-hepten-2-one	0.01
19	3-Nonanone	0.03
20	*n*-Hexanol	0.10
21	*n*-Tridecane	<0.01
22	Heptylmethyl ketone	0.02
23	*n-*Nonanal	0.02
24	3-Octanol	13.73 (**2,850.00 *μ*g/Kg**)
25	(2E)-Octenal	0.09
26	3,6-Dimethyl-3-octanol	<0.01
27	1-Octen-3-ol	0.08
28	*n*-Heptanol	0.01
29	Dihydromyrcenol	0.01
30	2-Ethyl hexanol	0.08
31	Hydroxymethyl 2-hydroxy-2-methylpropionate	0.01
32	(2E,4E)-Hexadienal	0.04
33	(2E)-Heptenol	<0.01
34	Benzaldehyde	2.34
35	Linalool	0.11
36	*n*-Octanol	0.07
37	*n-*Tetradecane	<0.01
38	2-Undecanone	0.03
39	(2E)-Octenol	0.42 (**654.00 *μ*g/Kg**)
40	1,5-Heptadiene-3,4-diol	0.03
41	Phenylacetaldehyde	0.46
42	*n*-Nonanol	0.05
43	(3Z)-Nonenol	0.05
44	Valencene	0.01
45	(Z)-*α*-Bisabolene	0.03
46	(E,E)-*α*-Farnesene	0.11
47	*n*-Decanol	0.01
48	Geranylacetone	<0.01
49	Benzyl alcohol	0.89 (**641.00 *μ*g/Kg**)
50	4-Methyl-1,5-heptadiene	<0.01
51	Phenethyl alcohol	0.08

**Table 3:** Validation data for predominant volatiles of *Agaricus bisporus*: linear regression data, limit of detection (LOD), limit of quantification (LOQ), and recovery values (average of three analyses).

Compound	Linear range (ppb) for DVB/Car/PDMS fiber	Linear range (ppb) for PDMS/DVB fiber	*R* ^2^	Regression equation	LOD (ppb) S/N = 3	LOQ (ppb) S/N = 10	Recovery (±RSD) % (10 ppb spiked)
3-Octanone	156.25–1,250.00	39.06–10,000.00	0.9959	*y* = 1657.7919*x*	0.8	3.2	98.2 ± 2.1
3-Octanol	78.12–2,500.00	39.06–10,000.00	0.9594	*y* = 276.52*x*	1.6	2.7	97.6 ± 2.3
Benzyl alcohol	78.12–625.00	78.12–2,500.00	0.9965	*y* = 106.81*x*	1.1	3.9	114.3 ± 3.2
(2E)-Octenol	156.25–1,250.00	78.12–10,000.00	0.9988	*y* = 60.664*x*	0.7	4.2	94.8 ± 1.1

## References

[1] Loria-Kohen V., Lourenço-Nogueira T., Espinosa-Salinas I., Marín F. R., Soler-Rivas C., de Molina A. R. (2014). Nutritional and functional properties of edible mushrooms: a food with promising health claims. *Journal of Pharmacy and Nutrition Sciences*.

[2] Yang N., Tong X., Xiang Y., Zhang Y., Sun H., Wang D.-C. (2005). Crystallization and preliminary crystallographic studies of the recombinant antitumour lectin from the edible mushroom *Agrocybe aegerita*. *Biochimica et Biophysica Acta*.

[3] Zhang M., Cui S. W., Cheung P. C. K., Wang Q. (2007). Antitumor polysaccharides from mushrooms: a review on their isolation process, structural characteristics and antitumor activity. *Trends in Food Science & Technology*.

[4] Fatmawati S., Kondo R., Shimizu K. (2013). Structure-activity relationships of lanostane-type triterpenoids from *Ganoderma lingzhi* as *α*-glucosidase inhibitors. *Bioorganic & Medicinal Chemistry Letters*.

[5] Beelman R. B., Royse D. J., Chikthimmah N. (2003). Bioactive components in button mushroom *Agaricus bisporus* (J. Lge) Imbach (Agaricomycetideae) of nutritional, medicinal, and biological importance. *International Journal of Medicinal Mushrooms*.

[6] Combet E., Henderson J., Eastwood D. C., Burton K. S. (2006). Eight-carbon volatiles in mushrooms and fungi: properties, analysis, and biosynthesis. *Mycoscience*.

[7] Splivallo R., Ottonello S., Mello A., Karlovsky P. (2011). Truffle volatiles: from chemical ecology to aroma biosynthesis. *New Phytologist*.

[8] Zheng R., Jie S., Hanchuan D., Moucheng W. (2005). Characterization and immunomodulating activities of polysaccharide from *Lentinus edodes*. *International Immunopharmacology*.

[9] Dinesh Babu P., Subhasree R. S. (2008). The sacred mushroom ‘Reishi’—a review. *The American-Eurasian Journal of Botany*.

[10] Petrova A., Alipieva K., Kostadinova E. (2007). GC-MS studies of the chemical composition of two inedible mushrooms of the genus *Agaricus*. *Chemistry Central Journal*.

[11] Kim K. M., Ko J. A., Lee J. S., Park H. J., Hanna M. A. (2006). Effect of modified atmosphere packaging on the shelf life of coated, whole and sliced mushrooms. *LWT—Food Science and Technology*.

[12] Guillaume C., Schwab I., Gastaldi E., Gontard N. (2010). Biobased packaging for improving preservation of fresh common mushrooms (*Agaricus bisporus* L.). *Innovative Food Science and Emerging Technologies*.

[13] Taghizadeh M., Gowen A., Ward P., O'Donnell C. P. (2010). Use of hyperspectral imaging for evaluation of the shelf-life of fresh white button mushrooms (*Agaricus bisporus*) stored in different packaging films. *Innovative Food Science and Emerging Technologies*.

[14] Akram K., Kwon J.-H. (2010). Food irradiation for mushrooms: a review. *Journal of the Korean Society for Applied Biological Chemistry*.

[15] Costa R. (2014). Newly introduced sample preparation techniques: towards miniaturization. *Critical Reviews in Analytical Chemistry*.

[16] Supelco Bulletin (2011). *A Practical Guide to Quantitation with Solid Phase Microextraction*.

[17] (2012). *FFNSC 2—Flavour and Fragrance Natural and Synthetic Compounds*.

[18] Adams R. P. (2007). *Identification of Essential Oil Components by Gas Chromatography/Mass Spectrometry*.

[19] (2014). *NIST Chemistry WebBook*.

[20] Joulain D., König W. A. (1998). *The Atlas of Spectral Data of Sesquiterpene Hydrocarbons*.

[21] La Chiusa L. (2008). *Il grande libro dei funghi d'Italia e d'Europa*.

[22] Acree T., Arn H. (2014). *Flavornet and Human Odor Space—Gas Chromatography-Olfactometry (GCO) of Natural Products*.

[23] Cenci R. M., Sena F. (2010). *Elementi chimici nei funghi superiori*.

[24] Mondello L., Costa R., Tranchida P. Q. (2005). Determination of flavor components in Sicilian goat cheese by automated HS-SPME-GC. *Flavour and Fragrance Journal*.

[25] Wu C.-M., Wang Z. (2000). Volatile compounds in fresh and processed shiitake mushrooms (*Lentinus edodes* Sing.). *Food Science and Technology Research*.

[26] Costa R., Tedone L., de Grazia S., Dugo P., Mondello L. (2013). Multiple headspace-solid-phase microextraction: an application to quantification of mushroom volatiles. *Analytica Chimica Acta*.

[27] Combet E., Henderson J., Eastwood D. C., Burton K. S. (2009). Influence of sporophore development, damage, storage, and tissue specificity on the enzymic formation of volatiles in mushrooms (*Agaricus bisporus*). *Journal of Agricultural and Food Chemistry*.

[28] Leguijt T., Yuksel D., van der Vuurst de Vries R., Royse D. J. (1996). Flavor and texture of the common mushroom *Agaricus bisporus*. *Mushroom Biology and Mushroom Products*.

[29] Wurzenberger M., Grosch W. (1984). The formation of 1-octen-3-ol from the 10-hydroperoxide isomer of linoleic acid by a hydroperoxide lyase in mushrooms (*Psalliota bispora*). *Biochimica et Biophysica Acta—Lipids and Lipid Metabolism*.

[30] Pennazza G., Fanali C., Santonico M. (2013). Electronic nose and GC-MS analysis of volatile compounds in *Tuber magnatum* Pico: evaluation of different storage conditions. *Food Chemistry*.

